# Multidimensional Sleep Problems as Mediators between Recession Hardships and Changes in Physical Health

**DOI:** 10.1093/geroni/igaf122.1784

**Published:** 2025-12-31

**Authors:** Julie Kirsch

**Affiliations:** University of Wisconsin–Madison, Madison, Wisconsin, United States

## Abstract

The 2008 economic Recession was associated with physical health declines and poor sleep outcomes. This study tests a novel composite measure of poor sleep as a mediator of Recession-related changes in physical health in a longitudinal sample of adults from the Midlife in the United States study (n = 2,674). Participants reported sleep problems across five dimensions (Regularity, Satisfaction, Alertness, Efficiency, and Duration) and functional health and total chronic conditions at pre-recession (2004-2006) and post-recession (2013-2014). Hardships included financial, housing, and job events attributed to the 2008 Recession. Adjusting for relevant covariates and pre-Recession sleep problems, regression models showed that recession hardships were positively associated with total post-Recession sleep problems (a = 0.05, 95% CI [0.03, 0.07], p <.001) and were positively associated with functional limitations (c = 0.03, 95% CI [0.02, 0.04], p <.001) and chronic conditions (c = 0.10, 95% CI [0.05, 0.15], p <.001). Further, post-recession sleep problems were associated with increases in functional limitations (b = 0.11, 95% CI [0.08, 0.14], p <.001) and chronic conditions (b = 0.48, 95% CI [0.38, 0.58], p <.001). The bootstrap estimate of the indirect effects of Recession hardships on health outcomes via post-Recession sleep problems were statistically significant (Functional limitations a*b = .004, 95%CI [.002, .006]; Chronic conditions a*b= 0.01, 95% CI [.006, .02]). More sleep health problems may be a key pathway through which Recession hardships lead to poorer health in middle and older adulthood and may be important targets for intervention.

